# Applying Interleaving Strategy of Learning Materials and Perceptual Modality to Address Secondary Students’ Need to Restore Cognitive Capacity

**DOI:** 10.3390/ijerph19127505

**Published:** 2022-06-19

**Authors:** Wen Chen, Chuansheng Chen, Baoping Li, Jiacai Zhang

**Affiliations:** 1 College of Teacher Education, Ningbo University, Ningbo 315211, China; chenwen1@nbu.edu.cn; 2 Advanced Innovation Center for Future Education, Beijing Normal University, Beijing 100875, China; 3 School of Artificial Intelligence, Beijing Normal University, Beijing 100875, China; 4 Department of Psychological Science, University of California, Irvine, CA 92697, USA; cschen@uci.edu; 5 Faculty of Education, Beijing Normal University, Beijing 100875, China

**Keywords:** direct attention, micro-lectures, interleaving effect, EEG

## Abstract

Online courses are prevalent around the world, especially during the COVID-19 pandemic. Long hours of highly demanding online learning can lead to mental fatigue and cognitive depletion. According to Attention Restoration Theory, ‘being away’ or a mental shift could be an important strategy to allow a person to recover from the cognitive overload. The present study aimed to test the interleaving strategy as a mental shift method to help sustain students’ online learning attention and to improve learning outcomes. A total of 81 seventh-grade Chinese students were randomly assigned to four learning conditions: blocked (by subject matter) micro-lectures with auditory textual information (B-A condition), blocked (by subject matter) micro-lectures with visual textual information (B-V condition), interleaved (by subject matter) micro-lectures with auditory textual information (I-A condition), and interleaved micro-lectures by both perceptual modality and subject matter (I-all condition). We collected self-reported data on subjective cognitive load (SCL) and attention level, EEG data during the 40 min of online learning, and test results to assess learning outcomes. The results showed that the I-all condition showed the best overall outcomes (best performance, low SCL, and high attention). This study suggests that interleaving by both subject matter and perceptual modality should be preferred in scheduling and planning online classes.

## 1. Introduction

The ability to focus attention on a task or lectures is very important for students [1]. Attention is however a finite and fragile resource, which becomes depleted over time [2]. The issue of attention has become particularly evident during the COVID-19 pandemic, because students have been forced to take courses online. Because online learning lacks face-to-face interactions that may help sustain students’ attention, teachers face a major challenge to keep students’ attention during class. One recently proposed solution is to use micro-lectures, i.e., 10- to 15-min multimedia presentations. This form of instruction has been popular in informal learning, but it has also been adopted by instructors in higher education and secondary schools in Mainland China, especially in combination with the flipped classroom approach [3]. Empirical research is needed to understand what types of instructional strategies during micro-lectures would keep students’ attention, lower their cognitive load, and yield high learning outcomes.

According to Attention Restoration Theory [2], there are two types of attention: voluntary or direct attention and involuntary attention. Whereas involuntary attention is automatically activated and effortless, direct attention is goal-directed and relies on self-control to steer away from distracting stimuli. Direct attention, however, is also fragile and needs to be restored. Researchers have explored various ways to change environments and experiences to help restore direct attention [4]. Attention Restoration Theory has identified four factors of environmental changes that are important to attention restoration, including being away (i.e., clearing the head from previous tasks), extent (i.e., the scope, content, and structure of the environment), fascination (i.e., the degree of the environment’s attraction to hold a person’s attention effortlessly), and compatibility (i.e., an appropriate fit between one’s motives or purposes and the environment). Of the four factors, three require changes to the physical environment and/or the students’ internal states, which are relatively difficult to accomplish during online learning, leaving only the “being away” factor as a practical way to restore attention. One “being away” or mental shift strategy is to interleave different learning materials, rather than to block the same materials together.

Interleaving means different types of class materials are presented in patterns such as “ABABAB”, rather than the traditional blocked order (e.g., the pattern “AAABBB”) [5]. Education researchers have recently investigated the effectiveness of interleaving different materials. A small but growing number of studies have found that interleaving has advantages over blocking in various types of learning, including sports [6,7], medical diagnoses [8], painting [9], mathematical learning [10,11,12], concept learning [13], and auditory perceptual learning [14]. A few studies have explored the benefit of the interleaved retrieval practice from the lab to the classroom [15,16]. Such empirical evidence has gradually challenged the time-honored tradition in education that classes are grouped in blocks.

Surprisingly, however, all those previous studies focused on interleaving by different types of skills or learning (e.g., problem solving or category learning) within a certain field rather than by perceptual modality (e.g., visual vs. auditory presentations), even though the use of multiple perceptual modalities has been emphasized by multimedia education (Mayer, 2009). Thus far, the interleaving effect across different modalities has been examined in only one study [17]. Szpiro et al. (2014) found that interleaving training trials of an orientation comparison task (target task, visual modality) with those of a spatial-frequency comparison task (interleaving task, auditory modality) improved the performance on the target task. One possible mechanism for the cross-modality interleaving effect is by reducing cognitive overload or slowing channel-specific resource depletion. Indeed, it has been found that a multi-channel presentation of stimuli (whether successively or simultaneously) led to reduced cognitive load, increased WM efficiency, and better learning outcomes [18].

Furthermore, few studies have examined interleaving by subject matter (e.g., reading vs. mathematics vs. history). Thus far, only one study has investigated the effect of interleaving by subject matter (language learning and biology facts), but it failed to find a beneficial effect [5]. One possible reason for the failure is that in that study (as well as all previous studies of the interleaving effect), interleaving occurred at the level of items or cognitive problems, which differed from the real-life learning of completing the learning of a concept such as in a micro-lecture. The current study examined the interleaving effect at the level of micro-lectures.

Specifically, the present study investigated the interleaving effect of subject matter (i.e., mathematics and history) and instructional modality (visual and auditory) on students’ attention level, subjective cognitive load (SCL), and performance. We used electrophysiological measures to assess sustained attention. Although students’ attention span in the classroom is often discussed in educational research, there is little classroom-based research on attention depletion of online learning [19]. The commonly held belief is that students’ attention span is 10–15 min [20], with limited evidence from self-reports [21], observations [22], and psychophysiological measures (e.g., heart rate) [23]. To the best of our knowledge, no neuroscience-based research has been conducted to investigate students’ attention in a regular class period. Different ways of measuring attention exist, including eye tracking, fMRI, EEG, and self-report. The present study used the portable EEG method, which has been proved to be a reliable way to measure attention in real-world learning situations [24,25,26] and self-reports of attention (i.e., the distracted time and SCL) [21,27]. Target performance was measured with tests based on the class materials.

Based on the limited existing research, we hypothesized that interleaving by subject matter (mathematics and history) and perceptual modality (visual and auditory) would lead to better attention (slower attention depletion), lower SCL, and better learning outcome.

## 2. Method

### Participants

Participants were 105 students from three 7th-grade classes of a high school in Beijing, China. The experiment was divided into two stages: a pilot study with students from one class (N = 34) and the formal experiment with the other two classes (N = 81). The pilot study was conducted to assess the levels of difficulty and discrimination of the quiz items for the five micro-lectures. Students of the other two classes were assigned into four groups by roughly matching their mathematics scores from the previous semester. Two students did not participate or finish the experiment. The matching was successful, as evidenced by non-significant differences in previous semester’s mathematics scores (*F* (3, 77) = 0.092, *p* = 0.964) and history scores (*F* (3,77) = 1.264, *p* = 0.291).

Students were not compensated for this experiment. Both the students and their parents signed informed consent forms after a full explanation of the study procedure. This study was approved by the Institutional Review Board of the School of Artificial Intelligence, Beijing Normal University Ethics Committee. All students and parents completed the signed informed consent form under the guidance the teachers in charge of the classes.

## 3. Materials and Tasks

### 3.1. Self-Reported Scales

After watching each micro-lecture but before taking the quiz, participants reported the amount of time they did not pay attention to (felt distracted from) the lecture on a 4-point scale: 1 = less than 10 s, 2 = 10 to 30 s, 3 = 30 to 60 s, and 4 = more than 60 s. At the end of the five micro-lectures and quizzes, participants were asked to assess SCL with 2 items (difficulty and mental effort) on a 9-point Likert type scale [28]: 1 = very easy or least effort, 9 = very hard or most effort. SCL was the sum of the two items.

#### EEG Data Acquisition

EEG was recorded by MindSet headsets during the learning process. The EEG included 8 bands of waves: delta (0.5–2.75 Hz), theta (3.5–6.75 Hz), low-alpha (7.5–9.25 Hz), high-alpha (10–11.75 Hz), low-beta (13–16.75 Hz), high-beta (18–29.75 Hz), low-gamma (31–39.75 Hz), and mid-gamma (41–49.75 Hz). Data were automatically corrected for eye blinks and ocular artifacts.

### 3.2. Experimental Materials and Tests

After surveying available resources for appropriate materials for 7th-grade Chinese students in their second semester, we selected three mathematics modules and two history micro-lectures as the learning materials. The format of teaching and the styles of the animations were well matched across topics (for more details, see Supplementary Materials).

Each of the micro-lectures was about 5 min long. Two versions of each micro-lecture were created: one auditory version (having sound but no subtitles) and one visual version (having subtitles but no sound). A quiz was created for each micro-lecture. Following Bloom’s Taxonomy of Learning Objectives [29], the quizzes included questions about knowledge, comprehension, and application. A pilot study was conducted to test the materials. In total, 34 students watched five micro-lectures and answered 36 corresponding questions. Six questions were found to be poor items (i.e., the criterion of item difficulty, P (proportion of students scored correctly) < 0.25 or >0.75; and discrimination, R (biserial correlation between item and total score) < 0.20). Of the final 30 items, there were 12 knowledge items, 13 comprehension items, and 5 application items. The three types of items were weighted as follows: a weight of 1 for knowledge, 2 for comprehension, and 3 for application. The theoretical range of scores was 0 to 53.

In the formal experiment, 81 students learned the micro-lectures and answered only the remaining 30 questions. The reliability (internal consistency) of the total test of the final 30 items was acceptable (*α* = 0.699). As evidence of validity, the score of this test was significantly correlated with students’ mathematics scores (*r* = 0.574, *p* < 0.001) and history scores (*r* = 0.678, *p* < 0.001) from the previous semester. During the experiment, EEG data and self-reported attention (or lack of it) and cognitive load were collected.

### 3.3. Experimental Procedure

**Figure 1:**
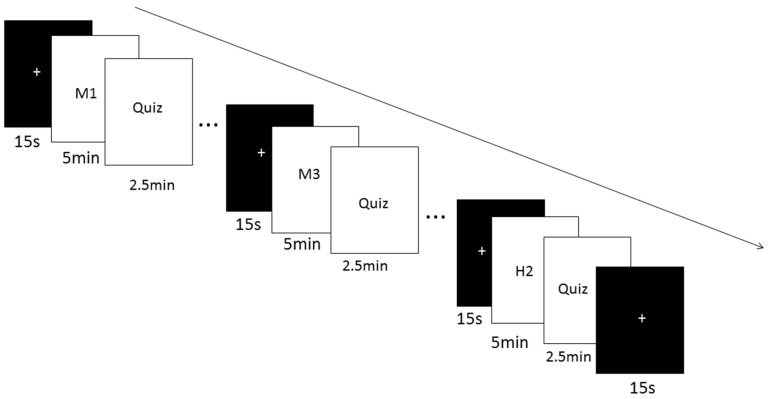
The experimental procedure of the formal experiment. The black screen with a white cross represents a 15 s rest. M and H mean mathematics and history micro-lectures, respectively. T means the test period.

Students used an online learning system to create an account and participate in the experimental tests. Figure 1 shows the learning process of micro-lectures and quizzes in alternating 15 s blocks of resting state. A brainwave-detecting headset was used during the formal experiment.

There were four learning conditions/groups in this study (see Table 1). The capital letters (M and H) indicate the version of lectures with no subtitles, and the lowercase letters (m and h) indicate the version of lectures with subtitles. Group 1 (the B-A condition, N = 20) learned micro-lectures blocked by subject matter (mathematics followed by history) with auditory instruction (no subtitles) (M1-M2-M3-H1-H2). Group 2 (B-V, N = 20) learned micro-lectures in blocks with only subtitles (no sound) (m1-m2-m3-h1-h2). Group 3 (I-A, N = 18) learned micro-lectures interleaved by subject matter with auditory instruction (M1-H1-M2-H2-M3). Lectures or micro-lectures are commonly presented aurally [30], so we only studied the interleaving subject matter condition with auditory instructions. Group 4 (I-all, N = 23) learned math micro-lectures interleaved by both subject matter and modality (m1-H1-m2-H2-m3).

Before the formal experiment, students were told that they would learn 3 mathematics and 2 history micro-lectures, take a time-limited (2.5 min) quiz after each video, and report their attention level and cognitive load. During the experiment, they would be wearing a brainwave headset. After the introduction, students were given a practice trial of a quiz to ensure that they understood the instructions. The whole experiment took about 40 min.

### 3.4. Data Analysis

IBM SPSS 19.0 was used to analyze the behavioral and EEG data. One-way ANOVA and ANCOVA were conducted to analyze the learning outcomes. Repeated measures ANOVA was used to analyze SCL and self-reported attention.

For the EEG data, the first 60 s were discarded, and the rest of the data were averaged at every time point (about one second) and then smoothed with a 15 s sliding window (86.67% overlap between successive windows). ANOVA was used to analyze both the average attention level during learning. Finally, we calculated the rate of change (ROC) in attention as the difference in attention level of one time point (a) and the beginning period (the first 15 s) of the next time point (b) divided by b: that is, ROC = (a − b)/b.

## 4. Results

### 4.1. Demographic Data

The mean age (and SD) was 12.20 ± 0.41 years for Group 1 (B-A), 12.33 ± 0.48 for Group 2 (B-V), 12.31 ± 0.47 for Group 3 (I-A), and 12.35 ± 0.49 for Group 4 (I-all). There was no group difference in mean age (*F* (3, 77) = 0.896, *p* = 0.447) and in gender composition (*χ*^2^ = 0.453, *p* = 0.929).

### 4.2. Test Scores

**Figure 2:**
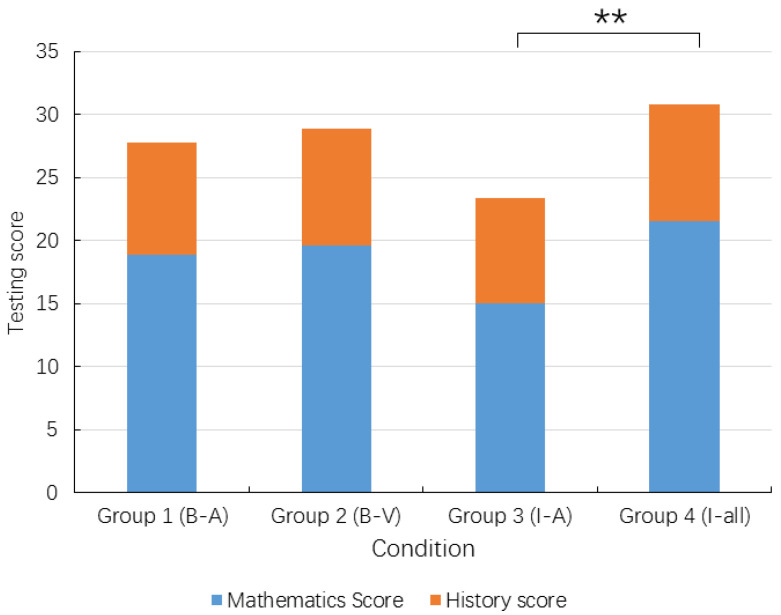
Group differences in test scores. B-A = blocked, auditory condition; B-V = blocked, visual condition; I-A = interleaved, auditory condition; I-all = interleaved auditory and visual condition. ** *p* < 0.01.

Figure 2 shows the means of the quiz scores (mathematics and history and their combined total scores) by condition/group. One-way ANOVA showed significant group differences in the total test score, (*F* (3, 76) = 3.463, *p* = 0.020, *η*^2^ = 0.122). After controlling for the students’ class performance (class test scores) in the two relevant subjects (mathematics and history), ANCOVA showed that group differences remained significant, (*F* (3, 76) = 2.747, *p* = 0.049, *η*^2^ = 0.099). Bonferroni *post hoc* analyses showed that the total score of Group 3 (I-A) was significantly lower than that of Group 4 (I-all). Although there were not enough items for separate mathematics and history tests, it is of some value to examine whether the group differences occurred in mathematics or history or both. Separate ANOVA (*F* (3, 76) = 3.100, *p* = 0.032, *η*^2^ = 0.110) and ANCOVA (*F* (3, 76) = 2.755, *p* = 0.048, *η*^2^ = 0.099) showed that group differences were significant for mathematics but not for history, which was perhaps due to the latter’s fewer items.

### 4.3. Subjective Cognitive Load

**Figure 3:**
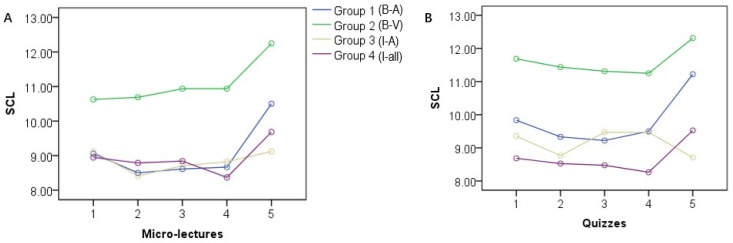
SCL during the five micro-lectures (**A**) and five quizzes (**B**).

The results of repeated measures ANOVA showed significant differences in SCL across the four groups both during micro-lectures (*F* (3, 65) = 2.706, *p* = 0.05, *η*^2^ = 0.111) and during quizzes (*F* (3, 65) = 3.50, *p* = 0.02, η^2^ = 0.139) (Figure 3A,B). Bonferroni *post hoc* test showed that the SCL of Group 2 (B-V) was significantly higher than that of Group 3 (I-A) and Group 4 (I-all) (*p* < 0.05) in quizzes but not in micro-lectures. The SCL of the fifth micro-lecture was significantly higher than that for other micro-lectures, and the same pattern was found for the SCL of the quizzes, except that the SCL of the first and fifth quizzes did not differ significantly.

### 4.4. Attention Level

Attention level was assessed via both self-reports and EEG. Self-reports of distracted time did not differ by group/condition or across learning and quiz periods. Here, we focus on the more sensitive EEG index.

**Figure 4:**
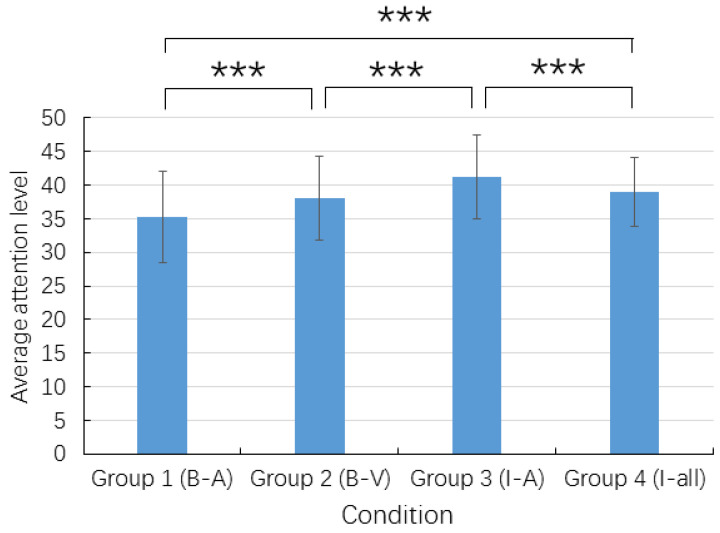
Average attention level by condition. *** *p* < 0.001.

**Figure 5:**
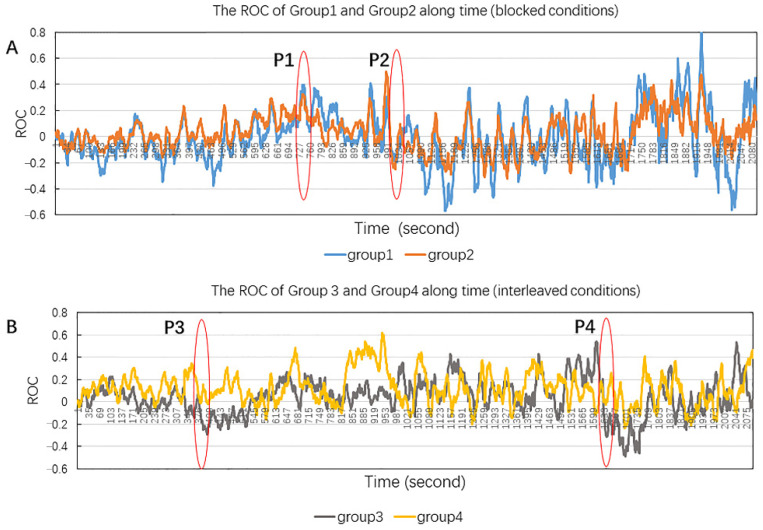
The ROC of average attention level of blocked conditions (**A**) and interleaving conditions (**B**) throughout the learning sessions. P1 is the early high point of blocked conditions; P2 is the turning point of attention going down as compared to the attention level at the beginning; P3 is the first point of time with the attention of Group 3 (I-A) going down; P4 is the turning point of attention declining for the interleaving conditions.

One-way ANOVA showed a significant group differences in the average attention level, (*F* (3, 8723) = 340.43, *p* < 0.001, *η*^2^ = 0.105) (Figure 4). Bonferroni *post hoc* test showed that all of the pairwise comparisons were significant for the four groups, with Group 3 (I-A) showing the highest attention. As Figure 5 shows, the two blocked conditions showed faster attention depletion than the two interleaved conditions. For the blocked conditions, ROC reached the first high point (P1) around 740 s (12.3 min) after the start of the micro-lecturers but declined somewhat afterwards and reached the second high point P2 at 1010 s (16.8 min), after which point, ROC reached below zero and fluctuated wildly afterwards (Figure 5A). The wild fluctuations indicated that the students’ attention was unstable and depleted. In contrast, the interleaving conditions did not show attention depletion at P4 around 660 s (27.6 min). It is worth noting that Group 3 showed a brief period (2.4 min) of attention decline at P3 (Figure 5B), which corresponded to the transition from mathematics to history. Group 3 regained attention quickly afterwards until P4.

We further investigated the group differences in attention level during micro-lectures and quizzes separately with two-way ANOVA. There were significant group differences in attention level both during micro-lectures (*F* (3, 5498) = 71.330, *p* < 0.001, *η*^2^ = 0.049) and during quizzes (*F* (3, 2949) = 413.326, *p* < 0.001, η^2^ = 0.296) (Figures S1 and S2). For the micro-lectures, Bonferroni *post hoc* test (*p* < 0.05) showed that both Group 3 (I-A) and Group 4 (I-all) showed significantly higher attention than Group 1 (B-A) and Group 2 (B-V). The third micro-lecture learning period showed significantly higher attention than other periods. For the quizzes, Bonferroni *post hoc* test (*p* < 0.05) showed that all of the pairwise comparisons across the four groups were significant, with Group 3 (I-A) showing the highest attention level.

## 5. Discussion

The current study investigated the interleaving strategy of subject matter and perceptual modality as a mental shift method to maintain students’ direct attention, to lower SCL, and to benefit learning outcomes. We found that (a) students’ attention started to decline after 10~15 min under the blocked conditions, (b) interleaving by subject matter alone led to the highest attention level but lowest performance, and (c) interleaving by both subject matter and perceptual modality led to better overall outcomes (high attention, low SCL, and highest performance).

Our EEG finding of short attention spans (about 10~15 min) during traditional blocked lectures is consistent with the consensus based on previous behavioral research [20]. The initial high and stable attention was then followed by great fluctuations between phases of attention and inattention [27]. With interleaving by subject matter and perceptual modality, students’ attention can be sustained, their SCL lowered, and their performance improved (the latter only for interleaving by both subject matter and perceptual modality, a point to which we will return). The results supported our hypothesis that interleaving is an effective method to restore attention. According to Attention Restoration Theory, interleaving means a mental shift from previous tasks, so the attention can recover. This finding of interleaving as an effective way to keep attention is particularly important, as other strategies, such as natural views [31,32], sufficient scope [33], and higher compatibility between a person’s motives and the environment [34] are relatively difficult to realize, especially in an online setting.

Researchers have provided three explanations for the interleaving effect. According to the discriminative-contrast hypothesis, the interleaved practice promotes the organizational and item-specific processing [11,35]. According to the retrieval-practice hypothesis, interleaved practice leads to distributed retrieval from long-term memory [36]. Finally, according to the attention attenuation hypothesis [37,38], it is more difficult to pay full attention to the subsequent materials when presented in blocks. For example, Carvalho et al. (2017) recently found that participants showed decreased overall memory and attention in the blocked condition as compared to the interleaved conditions [39]. Our results are consistent with the attention attenuation hypothesis, and they suggested that direct attention under the interleaved condition may be a main mechanism.

We did not find a significant performance difference between the B-A and B-V conditions. Previous research on the effects of subtitles has been mixed. On one hand, many studies have provided evidence that various forms of subtitles have significant potential in education [40,41,42]. On the other hand, it is common to find that subtitles showed either no positive effect [43] or even a detrimental influence [44,45] on student’s performance in second language studies. Our results belonged to the latter camp. Future research needs to specify conditions when subtitles help or interfere with learning.

Although interleaving led to higher attention and lower SCL, it did not always produce better performance. In fact, interleaving by subject matter alone led to the worst performance. One plausible explanation is the nature of the instruction materials. We used multimedia materials for our micro-lectures but limited the presentation only to auditory information for the I-A condition (as well as the B-A condition). It is possible that the reduction in information channels was hurting the I-A condition more because the auditory processing of information in two subject matters was more effortful than either under a blocked condition or with some assistance of visual information. As the test scores showed, the micro-lectures were relatively difficult to begin with (with means less than half of the total possible scores). Consistent with this expectation, the I-A condition indeed showed the highest level of attention. Future research should use materials with different levels of difficulty and particularly materials that are developed for auditory presentation to examine difficulty’s moderating role in the effects of interleaved instructions.

### Educational Impact and Implications Statement

The present study revealed that not all types of interleaving have positive effects. Whereas interleaving by both perceptual modality and subject matter in micro-lecture learning led to better learning outcomes (high attention, low SCL, and best performance), interleaving by subject matter alone led to high attention but worst performance. More research is needed to find the best combination of interleaving conditions.

## 6. Limitations

Several limitations of this study should be noted. First, for better control of materials and quality of presentation, we used micro-lectures instead of actual classroom instructions. Future studies could use the interleaving strategy of both learning materials and perceptual modality within one real classroom lecture or between lectures, but in a well-organized way to avoid disorder. Second, we did not investigate all possible combinations of conditions (condition by subject matter by modality by serial order). In addition to mathematics and history, it would be valuable to explore micro-lectures for other subjects to achieve a “mental shift” effect to restore students’ cognitive capacity. More forms of micro-lectures should be tested to optimize the multi-media resources. Third, the sample size of this study was relatively small.

## 7. Conclusions

This micro-lecture-based EEG study found that interleaving by both subject matter and perceptual modality slowed down attention depletion and led to better learning outcomes.

## Figures and Tables

**Table 1:** Four learning conditions/groups in the formal experiment.

Condition	The Number of Participants (Male/Female)	Learning Method	Textual Information Channel	Learning Order
Group 1 (B-A)	N = 20 (11/9)	Blocked	Auditory	M1-M2-M3-H1-H2
Group 2 (B-V)	N = 20 (10/10)	Blocked	Visual	m1-m2-m3-h1-h2
Group 3 (I-A)	N = 18 (8/10)	Interleaved	Auditory	M1-H1-M2-H2-M3
Group 4 (I-all)	N = 23 (12/11)	Interleaved	Visual & Auditory	m1-H1-m2-H2-m3

Note: M = Auditory mathematics, m = Visual mathematics, H = Auditory history, and h = Visual history.

## Data Availability

The data used to support the findings of this study are available from the corresponding author upon request (jiacai.zhang@bnu.edu.cn). All the participants have a key the number; therefore, the risk of identification is very low.

## Supplementary Materials

The following supporting information can be downloaded at: www.mdpi.com/article/10.3390/ijerph19127505/s1, Figure S1: The average attention level by condition and learning period during micro-lectures; Figure S2: The average attention level by condition and learning period during quizzes.
