# Supplementary material for: Applying Interleaving Strategy of Learning Materials and Perceptual Modality to Address Secondary Students’ Need to Restore Cognitive Capacity
Source: Int J Environ Res Public Health. 2022 Jun 19;19(12):7505. doi: 10.3390/ijerph19127505 (PMC9223479; doi:10.3390/ijerph19127505)

The Interleaving Effect of Subject Matter and Perceptual Modality on Students’ Attention and Learning: Evidence from Behavioral and Electrophysiological Measures

Supplemental Materials

Experimental materials

After surveying available resources for appropriate materials for 7th-grade Chinese students in their second semester, we selected three mathematics modules from "Onion Mathematics" (http://yangcong345.com/student.html) and two history micro-lectures from "Smart Learning Partner" (http://slp.bnu.edu.cn). The topics of the three mathematics micro-lectures were 'real number', 'square root' and 'arithmetic square root', which are highly related mathematical knowledge according to the syllabus of middle school textbooks published by the People's Education Press (http://www.pep.com.cn/). The format of teaching (first introducing the definition of the concept and then showing related calculations) and the styles of the animations were well matched across topics. The topics of the history videos included the introductions of the Sui dynasty and the Qing dynasty. Each topic included three main ideas and the style of the animations was again the same.

Figure S1. The average attention level by condition and learning period during micro-lectures. Bonferroni post hoc test ($p < .05$) showed that both Group 3 and Group 4 showed significantly higher attention than Group 1 and Group 2. The third micro-lecture learning period showed significantly higher attention than the other periods.

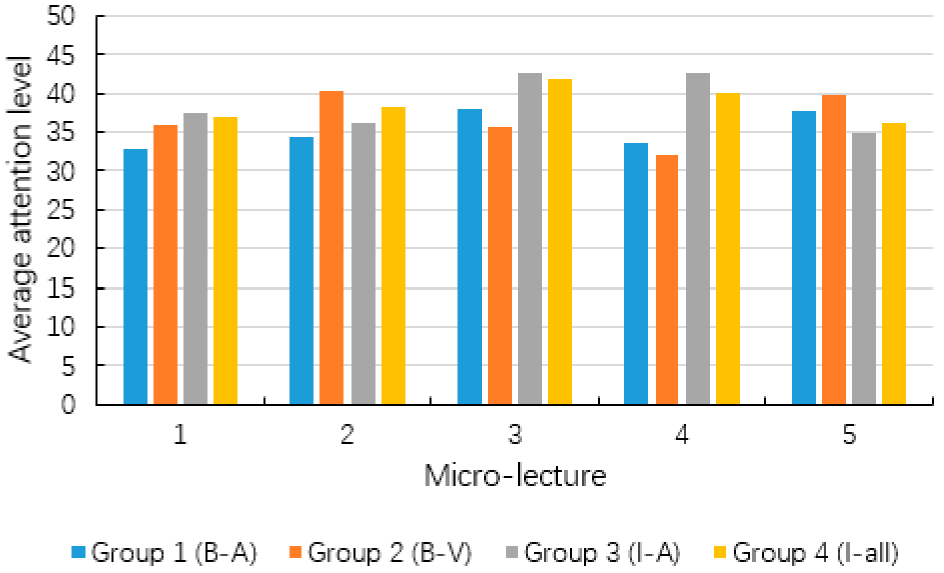

Figure S2. The average attention level by condition and learning period during quizzes. Bonferroni post hoc test (p < .05) showed that all of the pairwise comparisons were significant across the four groups, with Group 3 (B-A) showing the highest attention. The first and third quiz periods showed the lower attention level than the other three periods.

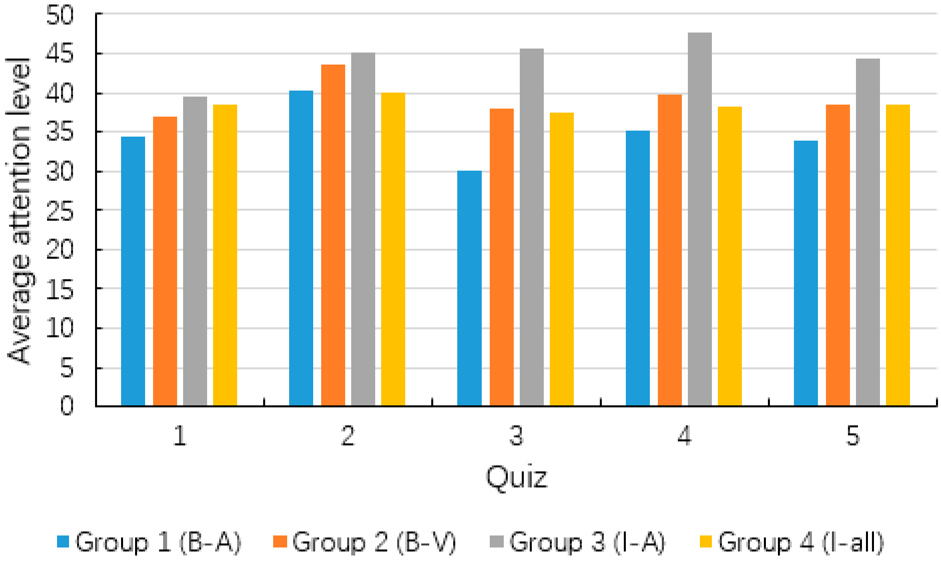

Supplement: Supplementary file 1 [file ijerph-19-07505-s001.zip › ijerph-1757420-supplementary.pdf]
